# Healthcare providers' perspectives on the organization of health services to manage people with multiple long-term conditions in primary care settings in Kerala, India: a qualitative exploratory study

**DOI:** 10.3389/fpubh.2025.1480710

**Published:** 2025-03-18

**Authors:** Thoniparambil Ravindranathanpillai Lekha, Linju Joseph, Neethu Vasantha Sasidharan, Athira Krishnan, Justine Davies, Paramjit Gill, Sheila Greenfield, Sivadasanpillai Harikrishnan, Jissa Vinoda Thulaseedharan, Mathew Joseph Valamparampil, Semira Manaseki-Holland, Panniyammakal Jeemon

**Affiliations:** ^1^Sree Chitra Tirunal Institute for Medical Sciences and Technology, Trivandrum, India; ^2^Institute of Applied Health Research, University of Birmingham, Birmingham, United Kingdom; ^3^Directorate of Applied Health, Warwick Medical School, Coventry, United Kingdom

**Keywords:** multiple long-term conditions, healthcare providers experiences, primary care challenges, non-communicable diseases, India, multimorbidity, primary care, health care providers

## Abstract

**Background:**

Multiple long-term conditions (MLTCs) are a major public health challenge globally. Complexity in managing MLTCs and their adverse consequences confronts the public healthcare systems in India. However, data from India to understand how to improve capacity to manage multiple chronic conditions are limited. We aimed to explore the challenges healthcare providers (HCPs) face in managing people with MLTCs in a south Indian primary care setting.

**Methods:**

Semi-structured interviews were conducted with HCPs in four districts of Kerala, India. Key themes and sub-themes were identified using the Framework method for thematic analysis. We categorized the systemic drivers that influenced management of patients with MLTCs in the government primary care settings as health system, organizational and individual HCPs, and patient-levels.

**Results:**

33 in-depth, semi-structured interviews were conducted. Two main themes with sub-themes were found: multimorbidity preparedness (*program and human resource planning; treatment guidelines and protocols; combination medicines; and handover communication between HCPs*), multimorbidity care *competence* (*awareness, implementation, and practices; attitudes of HCPs; and multimorbidity patient characteristics*). Management of MLTCs at primary care was facilitated by the presence of programs for chronic respiratory conditions and depression, perceived value of electronic health records, awareness of HCPs regarding programs and patients' needs. However, several challenges at the health system level including lack of long-term planning, treatment guidelines and combination medicines, leading to fragmentation of care and poor program implementation and uptake by HCPs and patients.

**Conclusion:**

Our study confirms sub-optimal health system preparedness and highlights the challenges for a transitioning primary care for managing people with MLTCs in one of India's states with a well-developed healthcare system. Our results suggest a need for improved planning and re-organization of primary health services with ongoing training support for HCPs.

## Introduction

Globally, the prevalence of multiple long-term conditions (MLTCs) has increased substantially in the past two to three decades and has substantive healthcare needs, stressing most health systems (1, 2). MLTCs or multimorbidity, refers to the existence of two or more long-term conditions in a single individual, which may be chronic non-communicable diseases (NCDs), chronic infectious diseases, and mental health conditions of long duration (3). The prevalence of MLTCs increases with several socio-demographic variables such as age and economic deprivation (4, 5). In low and middle-income countries (LMICs) such as India, NCDs are the most significant contributors to mortality and morbidity (6, 7), including an increasing prevalence of mental health disorders (8).

There is a lack of consensus regarding the most effective approach to managing care for people with MLTCs (9, 10). Further, the existing care models often prioritize addressing individual diseases in silos, with specialists for each disease rather than considering the comprehensive requirements and contexts of individuals with complex care needs (11, 12). Generally, people with MLTCs utilize healthcare services more frequently than those with a single disease (1). The long-term nature of these conditions requires patients to undergo frequent examinations, take various medications, and attend numerous medical appointments with different healthcare providers (HCPs), leading to fragmentation of care, which increases the treatment burden (13–15). The enormity of the burden, the complexity of managing MLTCs, and the worldwide diversity in primary care systems calls for developing contextually relevant, resource-sensitive, and culturally appropriate primary care models. This is particularly the case in India which has a growing number of people with MLTCs (16, 17).

In response to the growing burden of NCDs in India, several policy actions, such as a National NCD program, have been taken by the government for effective management in the public health system (18). However, several studies highlighted the significant gaps in the public health system to manage chronic conditions effectively (19–22). The state of Kerala in India (the focus of this paper) has implemented health system reforms to enhance primary care services in the public health system. These reforms include the introduction of diabetes and hypertension screening and treatment, the adoption of electronic health records, and the establishment of a structured sequence of checkpoints for patients, leading to improved patient flow at the family health centers (FHCs), which are upgraded primary health centers (PHCs) (23–25). Additionally, the Directorate of Health services in Kerala expanded primary care services by including SWAAS (*Stepwise approach to airway diseases*) for Chronic Obstructive Pulmonary Disease (COPD) management and ASWASAM for depression screening and management (23–25). However, how these changes address (or not) the needs of individuals with MLTCs is unknown.

There is limited research from India on MLTCs, and none examines the challenges for HCPs in managing MLTCs at the primary care level. In our first paper of the series, we published the perspectives of patients with MLTCs, who reported care coordination difficulties such as traveling to multiple healthcare facilities, leading to fragmented and reduced continuity of care, struggles with medication procurement and management, and primary care not being sufficient to meet their healthcare needs (26). To obtain a complete picture of the issues needing to be addressed for a health system model, in this paper, we explored the perspectives of HCPs in Kerala's public health system in managing MLTCs in primary care, and identified facilitators and barriers to care within the context of primary care reforms.

## Methods

### Study design

We conducted a descriptive qualitative study as part of the project Systems Thinking Approach to developing an Integrated and patient-centered intervention model for multimorbidity care in primary care settings in India (27) between June 2022 and February 2023 using a thematic analysis (the Framework method) (28).

### Settings

The study was conducted in eight FHCs in the Northern (*n* = 3), Central (*n* = 3), and Southern (*n* = 2) districts of Kerala. These settings were selected to represent different geographical regions of Kerala. As stated above, Kerala revitalized its primary health centers (PHCs), transforming them into patient-friendly FHCs as part of the “Aardram”, a mission-based initiative in 2016 (23–25). The initiative introduced a series of reforms in the state's health sector at the FHC level with extended hours of operation and improved quality and range of NCD services with the support of local self-government (LSG) or panchayats (decentralized local bodies). As early as 1995, Kerala had taken steps to fulfill the constitutional mandate for decentralizing power, following which the state transferred funds, functions, and functionaries from various state government institutions, including health, to LSGs. LSGs consist of a three-tier system of local government in Kerala, consisting of gram panchayats (village councils), block panchayats, and district panchayats (29, 30). Elected members of LSGs collaborate with FHCs' officials to assess community health needs and implement tailored strategies by using fiscal and administrative policies vested in the LSGs (31).

The upgraded FHCs are staffed by three medical officers (doctors), four staff nurses, two pharmacists, and one laboratory technician. The FHCs provide NCD preventative and curative health services including screening and management of diabetes, hypertension, chronic respiratory diseases, and depression (23–25).

### Study participants and sampling

All primary health centers upgraded to FHCs in the north, central, and southern regions were eligible to participate in the study. We aimed to recruit at least two centers from each region and selected FHCs based on their availability to participate. The study participants were HCPs. HCPs were purposively (32) sampled to include doctors, nurses, and pharmacists, working in the FHCs and hospital specialists. Hospital specialists working in the public health system (secondary or tertiary) were eligible to participate in the study and were purposively sampled to include specialists in medicine, pulmonology and psychiatry based on the corresponding specialty clinic services available at the FHCs. Researchers visited the FHCs to conduct the interviews and recruited until theoretical saturation (33) was obtained. Researchers contacted hospital specialists by phone and agreed interviews at their preferred location or by telephone (34). Face-to-face interviews took place in private rooms within the healthcare facilities to maintain confidentiality. Telephone interviews took place based on the convenience of the HCPs.

### Data collection

We collected qualitative data through in-depth semi-structured interviews with the study participants. Experienced qualitative researchers (LJ and LTR) led the data collection process, assisted by two research assistants (AK and NS). The interviews were conducted in English or Malayalam, the regional language, if preferred by participants, to ensure effective communication. Before conducting the interviews, the research assistants received specific training by conducting mock interviews using the topic guides. Topic guides (Supplementary Box S1) were piloted with three HCPs to ensure clarity. Topic guides were prepared based on an understanding of the literature on NCD management in India, MLTCs globally, and discussion with the research team. The topic guide was designed to explore the experiences and challenges HCPs face in managing MLTCs in primary care settings. The interviews were audio-recorded.

### Data analysis

Interviews lasted between 20 and 120 min; there was no difference in length of interviews between face-to-face and telephone. Three researchers (LJ, AK, and NS) transcribed and translated the interviews in Malayalam to English, and one researcher (LTR) crosschecked the transcriptions against the audio recording for discrepancies. The Framework method (28) was used to identify and explore key themes and sub-themes related to the experiences of all interviewees in providing care for patients with MLTCs.

Two researchers (LJ and LTR) independently carried out the open coding of a sample of transcripts. These initial codes and areas from the topic guide were discussed among the two researchers (LJ and LTR) to develop a coding framework for the analysis of the remaining transcripts. This coding framework was subsequently used to code the complete interviews with ongoing discussions and revisions (PJ, LJ, LTR) as required. Taguette (35), qualitative data software, was used to facilitate coding. After coding all interviews, the codes and interview data were placed into a Microsoft Excel matrix, sorted by participant identifier and code name. An iterative process was followed to develop categories by regrouping codes for detailed review and interpretation of the recurrent themes and sub-themes. Themes and sub-themes were reviewed by the whole team. To determine barriers and facilitators, the interview data within the themes and sub-themes were deductively organized under three headings health system, organizational and individual HCPs, and patient-levels. We have reported the qualitative study using SRQR (Standards for Reporting Qualitative Research) checklist (see Supplementary Table S2; Supplementary Box S2).

### Ethics

We obtained Institutional Ethical Committee approval for the study from the Sree Chitra Tirunal Institute for Medical Science and Technology, Thiruvananthapuram (IEC/1543). Written informed consent was obtained before data collection.

## Findings

We interviewed 10 doctors, 8 nurses, 5 pharmacists working in 8 FHCs in Kerala, and 10 hospital specialists working in secondary or tertiary settings in the public health system in Kerala (Table 1). They were interviewed either face-to-face (*n* = 27) or by telephone (*n* = 6). HCPs were 7 male and 26 female participants with work experience ranging from 6 months to 17 years from north (*n* = 8), central (*n* = 14), and south (*n* = 11) regions in Kerala.

**Table 1:** Demographic details of interviewees.

**Participant ID**	**HCP category**	**Gender**	**Years of experience**	**Institution location**
ID1	Doctor	F	13	South
ID2	Doctor	F	8	South
ID3	Doctor	F	0.5	North
ID4	Doctor	F	12	North
ID5	Doctor	F	5	North
ID6	Doctor	M	12	Central
ID7	Doctor	M	15	Central
ID8	Doctor	F	3	Central
ID9	Doctor	F	3	North
ID10	Doctor	F	4	South
ID11	Staff nurse	F	6	South
ID12	Staff nurse	F	6	South
ID13	Staff nurse	F	9	South
ID14	Staff nurse	F	4	Central
ID15	Staff nurse	F	15	North
ID16	Staff nurse	F	10	North
ID17	Staff nurse	F	4	Central
ID18	Staff nurse	F	15	Central
ID19	Pharmacist	F	10	South
ID20	Pharmacist	F	3	South
ID21	Pharmacist	M	17	Central
ID22	Pharmacist	F	4	North
ID23	Pharmacist	F	7	Central
ID24	Specialist-psychiatry	M	8	Central
ID25	Specialist-psychiatry	F	11	North
ID26	Specialist-psychiatry	M	10	Central
ID27	Specialist-psychiatry	F	7	Central
ID28	Specialist-medicine	F	8.5	South
ID29	Specialist-medicine	F	22	Central
ID30	Specialist-medicine	M	8.5	Central
ID31	Specialist-respiratory medicine	F	15	South
ID32	Specialist-respiratory medicine	M	26	South
ID 33	Specialist-respiratory medicine	F	6	Central

**Figure 1:**
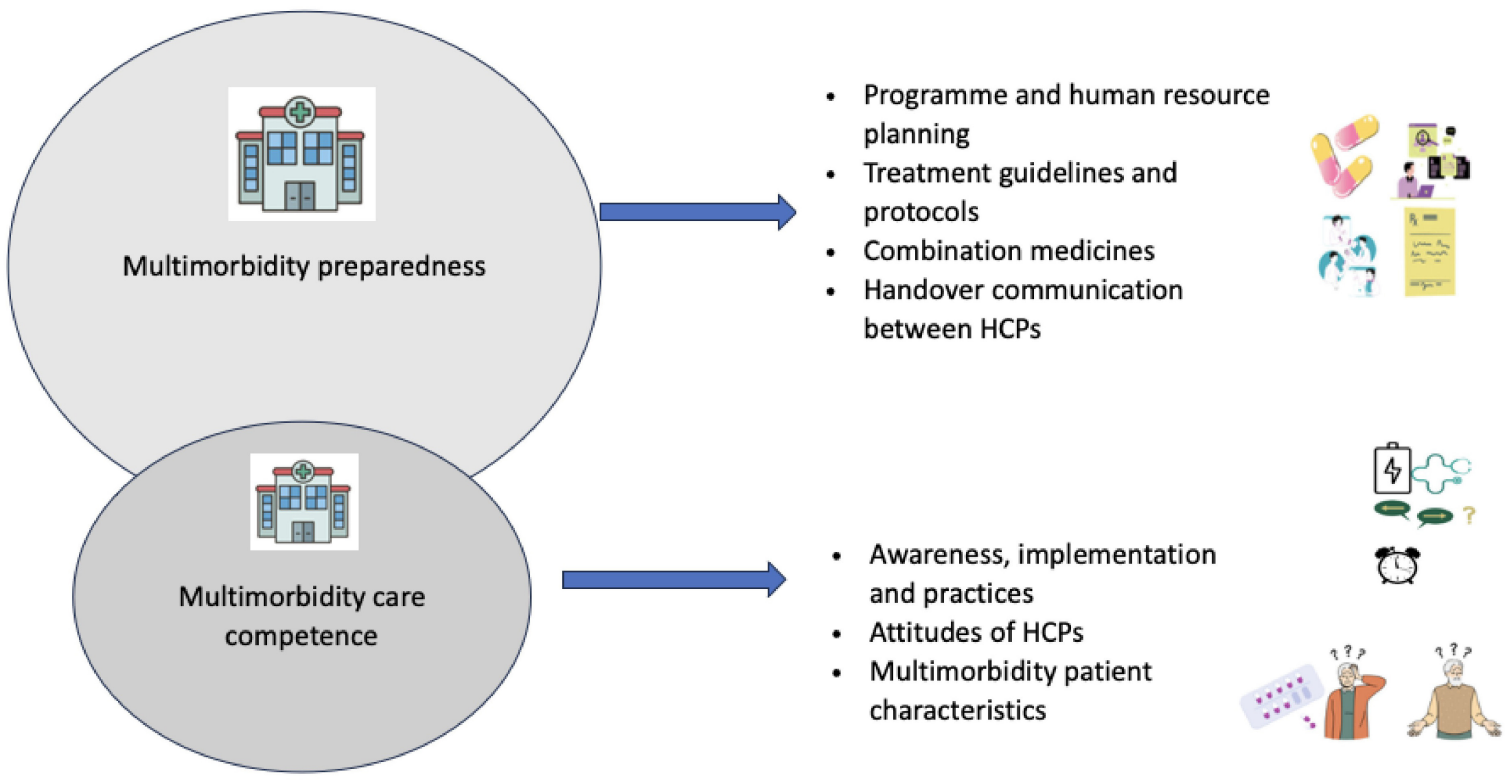
Themes and sub-themes illustrated.

The findings were organized into two main themes and sub-themes (see Figure 1). HCP quotes supporting the themes and sub-themes are illustrated within the manuscript. Additional quotes are presented in the Supplementary Table S1.

## Multimorbidity preparedness

Multimorbidity preparedness addresses the gaps and opportunities in managing people with MLTCs in the existing public health system. It is split into four sub-themes: program and human resource planning, treatment guidelines and protocols, combination medicines, and handover communication between HCPs.

### Programme and human resource planning

HCPs emphasized that there is a lack of long-term planning for programs and as a consequence patient-centeredness is not a key element in care delivery. They explained long-term planning issues related to deficiencies in fund allocation, several vertical programs leading to duplication of work and wastage of resources; and consequently, some stages of the program, such as screening, may function while others, such as treatment and follow-up often fail, leading to fragmentation of care.

“*We screen and find out all those with issues, but do not have a provision to give them medications or the doctors to treat them. In such a situation, the system will not or cannot take responsibility. When we start it as a new programme such as initiatives for chronic respiratory conditions, initially it will run with a high intensity such as screening activities but later the administrator's concern will be that it is not sustainable.” (ID32, Specialist, Respiratory medicine)*

“*Many agencies and vertical programmes doing the same work, means multiple diabetes detection camps and comorbidities detection camp. They will do all those that are meant for screening and detecting new cases, but our patients who are used to taking treatment from secondary and tertiary care centers will go directly, and will again get screened.” (ID29 Specialist, Medicine)*

Generally, doctors who have completed undergraduate training are posted at the primary care level. However, many doctors who have also completed specialty training work in FHCs. Some doctors working in FHCs with specialty training felt that their expertise could not be put into practice due to the hierarchical transfer of responsibility from primary care to higher centers for case management.

“*Specialists should be rearranged and posted in taluk or district hospital to improve the quality; otherwise, we will not be able to manage the patients with the limited resources at the primary care level even if we can.” (ID9, Doctor with specialty training in Medicine working at family health centre)*

### Treatment guidelines and protocols

Most doctors at FHCs responded that they have several cases of patients with MLTCs. Doctors reported that common NCD MLTCs are diabetes, hypertension, dyslipidaemia, chronic obstructive pulmonary disease, cardiac diseases, and osteoarthritis. Staff nurses and pharmacists reported similar conditions for patients with MLTCs visiting the FHCs.

“*Then I would say COPD cases and asthma along with diabetes and hypertension. Then joint pains, age-related all kinds of pain.” (ID2, Doctor with specialty training in Paediatrics working at family health centre)*

HCPs at FHCs described their current approach to managing patients with MLTCs. They mentioned adhering to treatment protocols for single-disease management (where available) and attempting to manage multiple conditions together. They explained how patients with MLTCs have several NCDs, however HCPs may not be able to provide comprehensive care for patients due to lack of services available at the primary care. Several HCPs reported the need to focus on non-pharmacological interventions such as diet, physical activity and stress reduction for preventing and managing NCD MLTCs. They emphasized that currently they provide incidental health education for patients with NCDs, but they also recognize that behavior change is challenging and needs supportive mechanisms for patients to adhere to the recommended lifestyle modifications which are currently not available.

“*So, for OA (osteoarthritis) knee, we can explain to them that painkillers are there. But they are only supportive management; physiotherapy and exercises are also part of it. We are not providing them at the primary care now. We do follow the STG (standard treatment guidelines).” (ID2, Doctor with specialty training in Paediatrics working at family health centre)*

“*Whether patients make changes in diet or increase physical activity or not, we tell them about it without fail. We cannot tell them they will be free from all diseases or that their diabetes and hypertension will vanish forever. Moreover, most people want to know whether they can stop their medicines if they start to walk. A few would also ask if they could escape from getting affected by diabetes or hypertension… That's the only thing we can do.” (ID4, Doctor working at family health centre)*

Most HCPs explained that having protocols and guidelines for managing patients with MLTCs particularly with emphasis on screening, early diagnosis and long-term care would be helpful. Both pulmonary and psychiatry specialists explained that they have developed guidelines for some specific conditions such as COPD, asthma, and depression. Two pulmonary specialists involved in the guideline development for COPD and asthma highlighted that they have included the common NCD comorbidities and their management. Specialists emphasized how the current system is highly single disease focused with several vertical programs running leading to missing comorbidities.

“*Whether the screening for diabetes or hypertension is done or not done, when the patient reaches the medical officers' OP (outpatient consultation), they will focus on the primary concern for which the patient has come, so patient may get the treatment for that disease and may miss out on the additional comorbidities.” (ID32, Specialist, Respiratory medicine)*

### Combination medicines

Pharmacists and doctors at FHCs raised concerns about the limited availability of combination medicines within the public health system. They also highlighted that the public health system may have only specific fixed dosages for many common drugs, which increased the number of pills patients had to take, complicating medication adherence.

“*The medicines prescribed for diabetic patients are single or fixed doses; they may need to take the same medicine twice or multiple pills to control their condition. Combination medicine is unavailable in cases like hypertension and diabetes in FHC.” (ID5, Doctor working at family health centre)*.

Further, HCPs at FHCs also highlighted the limited availability of medicines for several NCDs such as chronic kidney disease which affects many patients.

### Handover communication between HCPs

HCPs reported that the implementation of electronic health records in the public health system would be beneficial for them to view patients' records and thus aid in information transfer between healthcare visits. However, many HCPs at FHCs reported difficulties in using electronic health records and need help to adapt to these changes, and specialists find documentation often incomplete.

“*I think ehealth (electronic health records) is an advantage when it comes to old people, we will get their medical information once we check the health record. So even if they forget to bring all their past medical details in every visit, we will have some of the medical information in the system (electronic health records) because they come here regularly.” (ID8, Doctor with specialty training in ENT working at family health centre)*

“*…Along with screening for respiratory conditions, hypertension and diabetes were also supposed to be screened. When I open my patient's files, what I see is that BP is not recorded, and blood glucose is not recorded. When I view the records, these are not being measured nor documented and so it is not being implemented well at the primary care level.” (ID32, Specialist, Respiratory medicine)*

Participants highlighted a prominent issue, the need for more effective communication among different HCPs across the health system. This problem was observed in the communication between specialty doctors and primary care doctors. HCPs from primary care reported that this lack of communication worsened when patients went to other HCPs; especially to HCPs from private healthcare settings.

“*A problem here is that there is no proper system. They (patients) will go to any physician and get themselves treated. In such a situation, sometimes, we cannot give the medicines from here (FHC) that have been prescribed for them from elsewhere. Moreover, we will be unable to contact their doctor, and there will be a communication gap.” (ID7, Doctor with specialty training in Community medicine working at family health centre)*

## Multimorbidity care competence

Multimorbidity care competence describes the HCPs' reported knowledge, skills, and attitudes regarding managing people with MLTCs specifically with the changes implemented in primary care. It is split into three sub-themes: awareness, implementation, and practices; attitudes of HCPs and multimorbidity patient characteristics.

### Awareness, implementation, and practices

Participants were asked to reflect on their experiences with the current programs in FHCs and how they fit or not with management of patients with MLTCs. Most doctors and nurses were aware of the need for screening patients with diabetes and hypertension for early diagnosis of long-term complications such as kidney diseases.

“*The current rising epidemic is not infectious disease but non-communicable diseases. If kidney diseases are diagnosed earlier, we can delay the progression with medication rather than going to dialysis. Anyhow we may not be able to completely prevent the occurrence, but delay the onset of CKD, especially in diabetes patients. We can control and screen if they have CKD or liver diseases. There is no use once they reach the end stage.” (ID9, Doctor with specialty training in Medicine working at family health centre)*

They also highlighted that currently most patients with MLTCs would be diagnosed elsewhere and would come to FHCs for repeat prescriptions. Most doctors and staff at FHCs reported how comprehensive services are not available for patients with MLTCs and hence it is often difficult to track or follow-up.

“*What is happening now is that the patients come initially but after that they will not come for regular checkups correctly or may go to other centres (private) for treatment or they may not be taking treatment.” (ID7, Doctor with specialty training in Community medicine working at family health centre)*

Both hospital specialists and HCPs at FHCs reported delays and inadequacies in the training for HCPs thus impacting the running of specialized clinics at FHCs. Specialists noted inconsistencies in screening for respiratory and mental health issues at FHCs. These inconsistencies ranged from missing screening to over diagnosing or inappropriately diagnosing respiratory conditions. Additionally, they felt that bidirectional screening for patients with known diabetes or other long-term conditions are not routinely screened for associated comorbidities, leading to missed opportunities for early intervention.

“*Here, the first thing needed is to identify that the patient is anxious or is having depression. And how much of this is identified in the primary care setting, I do not know.” (ID31, Specialist, Respiratory medicine)*

Most HCPs from FHCs in this study reported having received training for running specialized clinics. However, most felt they were not sufficiently equipped to carry out the screening.

“*Two of our staff nurses have received training for the fundus test. But they are not confident enough to handle it themselves.” (ID7, Doctor with specialty training in Community medicine working at family health centre)*

Nurses also reported varying practices in screening patients referred for sleep or emotional difficulties, or mental health conditions.

“*No, we don't use any [assessment] scales to measure or the questionnaire; we just talk to them, and if we feel like they need assistance, we will provide that.” (ID16, Staff nurse working at family health centre)*

Even with additional HCPs at FHCs, HCPs reported limited time for interactions with patients at the primary care level, hindering effective management of patients with MLTCs.

“*Usually, screening time is significantly less in the periphery. We can detect multimorbidity in the first instance itself but the amount of time available to a primary care physician for screening is less. There is a mismatch in the patient load and time available.” (ID 28, Specialist, Medicine)*

### Attitudes of HCPs

Within the background of HCPs reporting no protocols or guidelines or provisions for managing patients with MLTCs in Kerala, when asked how to improve care for patients with MLTCs in primary care, they articulated organizational boundaries for responsibilities and tasks at the FHC level which suggests that primary care may not be suitable for managing patients with MLTCs. As a result, they believed that providing care for individuals with MLTCs at the primary care level is not feasible. They asserted that implementing additional services at the FHC is necessary if they must manage MLTCs, which would, in turn, require increased staff. Some HCPs felt that managing complex MLTCs should not be the responsibility of primary care.

“*If we added an ECG setup but did not have enough staff to manage it, we can refer them to a centre where the facility is available. Therefore, there will be an unnecessary delay, or we will need to have more and more necessary support factors related to it (screening or support activities), like more staff, facilities, admits, observation, etc. The patients may feel comfortable with this, but by starting one service at the FHC level, we will need to arrange more related facilities. Honestly, I am not convinced we need to arrange more facilities like that.” (ID4, Doctor working at family health centre)*

Doctors at FHCs emphasized a prevailing organizational culture encouraging patient referrals to specialists or higher centers with better facilities. They cited past experiences and saw it as a risk to treat these patients, especially in emergencies. Furthermore, doctors pointed out that patients with MLTCs often prefer consulting their specialists when deciding on medications or titrating the doses or further treatment plans.

“*After any cardiac intervention, it is unlikely that patients will continue coming here; they will most likely revert to their previous specialists. Besides those who need regular medication or BP/sugar check-ups, very few patients rely on us. Additionally, post-intervention, some patients may experience further symptoms, and as a primary care facility, we cannot afford to take unnecessary risks. That is why we often refer them to a cardiology specialist. It is worth mentioning that even the general hospital lacks a cardiologist. When higher-level facilities are hesitant to take risks, it becomes challenging for us at the primary care level to do so.” (ID9, Doctor with specialty training in Medicine working at family health centre)*

Particularly with respect to mental health conditions, specialists reported that doctors in primary care were not inclined to screen for them in patients with MLTCs even with the guidelines. Hospital specialists and doctors at FHCs reported that they try to refer patients with MLTCs, especially when the patient has had a history of previous cardiovascular intervention or when the patient has psychiatric conditions, as they do not feel obliged to manage them.

“*I have completed MD in ENT surgery. Many people with psychiatric-related symptoms come to our FHC. It can be difficult to handle people with mental health problems; we cannot change the dosage of their medicines or anything like that. We can give them the little help they need by repeating the medicines. Those who require counselling are usually referred for a psychiatric consultation.” (ID8, Doctor with specialty training in ENT surgery working at family health centre)*

Several doctors at FHCs reported actively liaising with the local self-governments for adding services for patients with NCDs including screening for thyroid conditions, medication support for patients on dialysis and secured provisions for gym equipment. However, they pointed out that these additional services are not sustainable without regular funding and therefore they often feel demotivated to work within primary care.

“*This time we have organised a diabetic neuropathy follow-up project at a panchayat level which includes screening camps for neuropathic complications but only if it is at government level, there is consistency in funding and we can provide services that patients need, or else it is certainly disappointing that we cannot continue the work we started.” (ID6, Doctor working at family health centre)*

### Multimorbidity patient characteristics

HCPs at FHCs pointed out that managing most patients with MLTCs is difficult because these patients often have many symptoms and are usually older, which brings additional challenges. Both doctors and nurses have emphasized that many patients, particularly older individuals with MLTCs, encounter challenges being alone at home. These difficulties mainly revolve around their ability to manage medications and adhere to prescribed diets. Being alone at home may lead to forgetfulness, confusion, or lack of support. Their medical conditions may restrict their ability to travel, leaving them with limited activities.

“*Mostly older adults, all they need is emotional support. And they are sad when it comes to having to deal all their different conditions.” (ID16, Staff nurse working at family health centre)*

Most HCPs noted that there is poor awareness regarding complications of diabetes and hypertension. They felt that if people are being guided and supported to manage lifestyle, medicines with adequate monitoring and follow-up, MLTCs may be delayed.

“*The problem with the younger age group is that they are not aware of complications. Actually, we should make them comprehend the consequences. See, life is not what we see or enjoy in the next 5, 6 or 7 years it is something which lasts longer. If a person loses his ability to see or has erection issues, then what will be his quality of life after that? or any kidney issues and then life-long dialysis. What will happen to his family?” (ID7, Doctor with specialty training in Community medicine working at family health centre)*

HCPs noted that the role of primary care could have been better understood by patients; especially their role in monitoring for complications, delaying progression to multiple conditions and ongoing management of MLTCs. Most HCPs reported that follow-up and monitoring for further complications was poor among younger patients and that younger patients stop medicines when the blood reports are normal. However, they felt that older patients were aware of the facilities available in the FHCs and sub-center level and would visit the facilities for monitoring their blood glucose and blood pressure.

“*Once they (patients) test and if their BP is normal, they decide to stop taking the medicine. For cholesterol, the same thing happens. If the value is less than 200, they will stop taking the medicine.” (ID23, Pharmacist working at family health centre)*

HCPs were aware that patients with MLTCs have a lot of issues that lead to non-adherence to medications. They pointed out that there were many gaps in the care system and felt that there is a need for improving the support systems for patients with MLTCs. HCPs pointed out financial difficulties as a reason for non-adherence, noting that medications for patients with MLTCs may not always be accessible even within the public healthcare system. Further, HCPs suggested that the patients often perceive the quality of medications provided by the public health system as inferior, further contributing to non-adherence to prescribed treatment regimens. Additionally, most doctors responded that the medication adherence issue is more when the patients are younger as they may have difficulty accepting that they are sick.

HCPs felt that younger and older people with MLTCs have difficulty adhering to recommended lifestyle modifications. Many HCPs recognized and felt that most people with MLTCs are under immense stress and need better support for managing lifestyle. They highlighted how younger adults are more stressed due to their work environments, while young and older patients are stressed regarding financial difficulties in daily life. They also noticed that most stress in people with multiple conditions is not managed well, leading to long-term health issues.

“*Younger age group would show reluctance in taking medicines and not only that they will not have any diet control and suffer from excessive stress and no lifestyle modification. What we can do is provide some advice. We tell them what we think they can do at home. For example, to walk at least 30 minutes, but right now we can only tell them.…they may need more individual support to plan and perform these self-care activities.” (ID5, Doctor working at family health centre)*

## Barriers and facilitators for managing MLTCs in primary care

**Figure 2:**
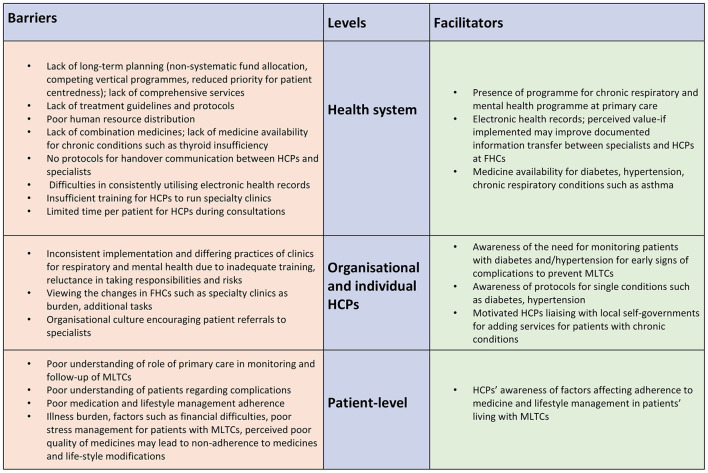
Barriers and facilitators for managing patients with MLTCs in Kerala.

The barriers and facilitators for managing MLTCS in primary care were organized under three levels; health system, organizational (at the primary care level) and HCP, and patient (see Figure 2). Overall, most barriers were classified at the health system level. The presence of a program for chronic respiratory conditions and mental health conditions at the primary care level and the ongoing implementation of electronic health records were facilitators for management of patients with MLTCs in primary care. Barriers at the health system level include poor planning, lack of treatment guidelines and protocols, lack of combination medicines and little or no protocols for communication between HCPs at the primary care and district/medical colleges. At the organizational and individual HCP level, HCPs' awareness regarding the need for monitoring for complications and liaison with local self-governments to organize screening and medications for long-term conditions such as thyroid, chronic kidney disease were identified as facilitators. Inconsistent implementation of specialty clinics, perceived confidence issues in implementing the screening due to insufficient training, attitudes toward screening and managing mental health conditions and reluctance to manage medications for patients with MLTCs indicated barriers to care. At the patient level, HCPs' awareness of the reasons for patient non-adherence and difficulties in management of lifestyle particularly due to financial difficulties and stress was a facilitator in managing care. HCPs identified the barriers of managing patients with several symptoms, patients' reliance on specialists and poor medication and lifestyle management.

## Discussion

Our study represents one of the first qualitative reflections of the perspectives of HCPs in India in managing patients living with MLTCs in primary care settings in Kerala, India. The study included the perspectives of specialists, doctors, nurses, and pharmacists regarding the management of patients living with MLTCs at primary care and can assist in informing development of this evolving healthcare system. The emergent findings were grouped into two main themes; multimorbidity preparedness, and multimorbidity care competence and the barriers and facilitators were organized under health system, organizational and individual HCPs, and patient-levels. Overall, most barriers were identified at the health systems level which hindered subsequent management of patients with MLTCs at the organizational and HCP level.

The HCPs highlighted several barriers at the health system level, including poor planning, lack of treatment guidelines, inadequate communication with other HCPs, and human resources, which collectively hinder the comprehensive and patient-centered management of patients with MLTCs. Our study acknowledges initiatives like the National Non-Communicable Disease (NCD) program in 2012 (36) and subsequent health sector reforms like Aardram in 2017 (23) in Kerala which aimed to enhance primary care services. However, our findings suggest that as well as control of conditions remaining a challenge, that health systems and HCPs continue to focus on achieving control for individual conditions, particularly (CVD, diabetes, respiratory illnesses) rather than addressing the broader challenges faced by patients with MLTCs. Further, our study shows that the exclusion of several chronic conditions such as musculoskeletal, neurological conditions and chronic kidney diseases have failed to address much of the NCD MLTCs' burden among the poor. This has been acknowledged as a limitation to global NCD strategies with the focus on prevention and management of selective NCDs (37, 38), our study shows how HCPs struggle in providing patient-centered and comprehensive care.

Previous research has highlighted a need for treatment guidelines for managing MLTCs globally (39, 40). Our study results suggest that health systems in Kerala and similar environments need to evolve to respond to the needs of HCPs to equip them to manage care for patients with MLTCs. Other studies have clearly stated that health service delivery should be guided by treatment protocols considering the potential interplay of multiple chronic conditions throughout the entire process, from diagnosis to management (41, 42). Along with an environment that enables the delivery of quality health care, our results also suggest the need for prioritization of the needs of individuals with MLTCs in the existing primary care guidelines and policy documents. The average consultation length in primary care settings is an essential determinant of quality of care as reported by Kruk et al. (43). Substantial evidence from clinical trials also supports longer consultations to improve the quality of life in individuals with MLTCs (44). While prioritizing care delivery for individuals with MLTCs in primary care, policy documents and guidelines should recommend reasonable consultation length.

Electronic medical records systems are considered a key facilitator for managing MLTCs. Available evidence supports the use of electronic medical records in care coordination (45). It promotes the quality and safety of patient care and improves the efficiency of HCPs' time and resource use, especially in managing chronic conditions. However, the introduction of electronic medical records in the primary care system needed to be better received by some HCPs in our study. Frequent disruptions in internet availability, lack of familiarity, the longer learning curve to use electronic medical records, and high patient load were cited as the main reasons for reluctance to use electronic medical records which are similar to previous findings (46, 47). Investments in improving the infrastructure and sufficient training may help the HCPs to adopt the electronic medical records system for efficient use of their time while managing chronic conditions.

Globally, failure to successfully implement and sustain change over the long term remains a major problem in primary care. Modifications made to routine clinical practice are known to be complex, and for them to be sustained over time, HCPs' behavior needs to change accordingly (48). Programmes such as ASWASAM trains doctors and staff nurses to provide psychosocial counseling and clinical guidelines for screening and management of depression. However, as found in this study, these are not easy to be adopted by HCPs. Interventions that aim to reorganize and strengthen new behavioral norms and connect them with the actions of peers and reference groups, such as opinion leaders, educational meetings with guidelines, and reminders for HCPs, are more likely to result in changes in behavior (49). However, these changes in clinical practice guidelines such as those envisioned in the ASWASAM and SWAAS, need to be well-supported with training, reminder systems and collaboration with specialists.

Our study revealed that the HCPs at FHCs interviewed relied on hospital specialists to manage patients with MLTCs. Lack of confidence in managing complex cases, training deficiencies and patients' preferences for specialist care were the primary reasons for referral to specialists. Findings from our qualitative study with patients with MLTCs also confirm that patients prefer hospital specialists to manage their multiple conditions (26). Globally, there have been difficulties for primary care providers regarding clarity in their role in screening and managing medications for patients with MLTCs (50, 51). In Kerala, where private and public specialists (52, 53) are available for providing care, our study emphasizes the need for a shift in the mindset of primary care providers, specialists and patients in managing MLTCs given that Kerala is one of the states with highest out-of-pocket expenditures for healthcare (54). Along with preventing chronic NCDs, primary care should ideally play a prominent role in monitoring and managing the complexities of patients with MLTCs (42). Collaborative interventions (9) that enhance communication between primary care providers and specialists for deciding management plans for patients with MLTCs must be explored and implemented. Generalists, medical officers (non-specialists), nurses, and pharmacists with adequate training can increase the coverage and ensure the quality of primary care delivery for individuals with MLTCs in LMIC settings.

Healthcare providers in this study identified challenges related to patient behaviors, such as lack of medication adherence, loss of follow-up, and difficulties in lifestyle management. From the HCPs' point of view, having combination medicines could help patients in adherence to medications. This is in line with the World Health Organization's recommendation of adding a polypill or fixed-dose combinations of multiple drugs for prevention and management of cardiovascular diseases to the World Health Organization Model List of Essential Medicines (EML) (55).

## Strengths and limitations

Our study provides critical insights into the LMIC perspectives on challenges faced by HCPs in primary care for managing MLTCs. Furthermore, the Kerala context adds value to the literature by exploring the health system challenges of managing MLTCs in a transitioning primary care system. Despite introducing health sector reforms recently in Kerala to manage NCDs in primary care effectively, the HCPs' perceptions indicate sub-optimal health system preparedness in managing MLTCs. Our study stands out as one of the few qualitative studies (56, 57) that have delved into HCP perspectives on the care they provide to patients with MLTCs within such a setting. We have selected HCPs from three different Kerala zones, improving the findings' possible transferability. A potential limitation is that while we have managed to gather perspectives of HCPs from primary care centers upgraded to FHCs, the FHCs could be in separate phases of upgrading. Therefore, HCPs' views on resources would have reflected the transition stage of upgrading primary health centers to FHCs. However, this is an actual representation of changes happening within the health system.

## Conclusion

Our study findings highlight substantial barriers at the health system level, including the need for treatment guidelines, inadequate communication among HCPs, and limited resources, which hinder the comprehensive management of patients with MLTCs in primary care in Kerala. These barriers highlight the need for further research that considers the interconnected relationships and dependencies within the health system. Addressing the systemic issues, rather than focusing on isolated components, can help avoid unintended consequences and achieve a more effective and integrated management of MLTCs. Group model building could be used to develop a shared understanding of interconnected factors influencing health system performance and access a wider range of potential leverage points for intervention. Hence, by developing an understanding on how positive outcomes are consistently achieved, we can design and implement intervention models that enhance overall system performance and ensure better care for patients with MLTCs.

## Data Availability

The original contributions presented in the study are included in the article/Supplementary material, further inquiries can be directed to the corresponding author.
