# Supplementary material for: Healthcare providers' perspectives on the organization of health services to manage people with multiple long-term conditions in primary care settings in Kerala, India: a qualitative exploratory study
Source: Front Public Health. 2025 Mar 18;13:1480710. doi: 10.3389/fpubh.2025.1480710 (PMC11960498; doi:10.3389/fpubh.2025.1480710)
Supplement: Supplementary file 1 [file Table_1.docx]

Online supplement

Table S1: Additional quotes

| Themes | Sub-themes | Quotes |
| --- | --- | --- |
| Multimorbidity preparedness | Programme and human resource planning | *If we have to implement something we have to consider the fund issues, not just the medicines. If we are giving care for mental health in the primary care, it should be consistent otherwise it is not helping the patients in anyway. (ID27, Specialist, Psychiatry) ￼* |
|  |  | *If you see in Kerala, many specialists are working in the PHC because of lack of specialty posting. Specialist availability is not an issue; we just need a system modification. If you post a specialist in an FHC or PHC, he or she cannot give specialist care there. Anyway, he is given salary by the system, if we utilize them well for the particular purpose they are trained for, it will be beneficial to both system and the patient, and we must make a system. (ID32, Specialist, Respiratory medicine)* |
|  |  | *I usually cannot do the patient counselling because we have a high number of patients. As per the current Indian standard a pharmacist needs to do 112 prescriptions (per day), here I do more than that, so patient counselling is not practical here. Manpower or workforce has to be increasedfor patient counselling needs, to be practical, for that at least three to four people must be there. (ID21, Pharmacistworking at family health centre)* |
|  | Treatment guidelines and protocols | *For hypertension and diabetes separate guidelines are available. A separate protocol is available in NCD guidelines. When we talk about multimorbidity, we are combining everything and giving the treatment. (ID1, Doctor working at family health centre)* |
|  |  | *It is hard to keep or maintain sugar levels or adequate control for diabetic patients; they are prone to hypertension, and patients have a tendency to skip or stop medicines and might have other health conditions as well. So, the control may not be achieved due to another condition. (ID6, Doctor working at family health centre)* |
|  | Combination medicines | *You might know that in our health service combination drugs are extremely limited. That is the problem. Those with hypertension, diabetes, COPD, and CAD, will nearly have 10 drugs to have. So, this problem (polypharmacy) may arise. But most patients are having medicine, but it is definitely a problem. If combination drugs were available, then it would have been good. (ID1, Doctor working at family health centre)* |
|  |  | *I think it (medicine list) would have been last revised 5 or 6 years back. Those protocols have no combination drugs for NCDs. As I said, in multiple conditions there will be multiple drugs, we are delivering only single condition drugs. Also, we will have only some fixed doses, so the patient may end up taking 4 tablets instead of one 1000mg tablet. When they (patients) visit private hospitals, they will mostly get combination drugs. Some patients used to come and ask if they want drugs they received from the private sector. Most of the patients here are from the poor socioeconomic background, so they will take whatever we provide. (ID22, Pharmacist)* |
|  | Handover communication between HCPs | *We are giving prescriptions now. E-health is paperless, but now we are giving prescriptions because when new patients come, the network may not be available, making it difficult. Network issues lead to losing all the data we typed so far, reducing the time to interact with the patients. In private institutes, we know they have a person to assist them in entering the details, but doctors in the public sector suffer a lot due to this problem. Even the doctor-patient ratio is much lower in the private sector. (ID1, Doctor working at family health centre)* |
|  |  | *Either they do not consume medicines, or they consume two tablets instead of one. I have been telling patients very clearly about the difference between each tablet, I tell them, I write them down, but still, patients mismatch, misplace, or skip. While I try to tell them in the busy OP, it is not always possible for me to know whether they have understood all that I am saying. (ID3, Doctor with specialty training in anaesthesia working at family health centre)* |
| Multimorbidity Care Competence | Awareness, implementation, and practices | *That (screening) applies to other ways also. A diabetic patient can have COPD, or a person with heart disease can have COPD. Reverse screening should be done to diagnose them, which is mainly missed. (ID32, Specialist, respiratory medicine)* |
|  |  | *Another fact is that the majority may not think of such an aspect, i.e., awareness among health professionals. If a patient is having anxiety or depression along with COPD, or asthma with diabetes or hypertension, 80% of them are often identified when the patient speaks about that. It is rare that we ask the patient in consultation and try to identify it. (ID31, Specialist, respiratory medicine)* |
|  |  | *If it is MI patients, if it is complicated, it will be difficult. Since FHC are open only till 6 pm it is even more difficult. Sometimes CVA patients will come, and then we will do the primary care and will refer them to the hospital. (ID13, Staff nurse working at family health centre)* |
|  |  | *We do not screen everyone; only if the doctor sends them to us will we screen them. They must have explained some issue, so they are being sent here for screening. We will refer them if the score is high in the first case itself. Otherwise, we have some medicines available here, which will be managed with that. (ID11, Staff nurse working at family health centre)* |
|  | Attitudes of HCPs | *Similarly, as we discussed about cardiac patients before like about having ECG facility. Even if ECG or ECHO facility is brought here, there will not be much use of it. We will not be able to manage it. However, if such care that of a physiotherapist is implemented it would be better. (ID4, Doctor working at family health centre)* |
|  |  | *We have basic investigation facilities for collecting serum creatinine, blood, and urea here in our lab. Almost all necessary tests are being done in our lab. But when we see the result and find the serum creatinine is high and hypertension is also not reducing then we could only guide them to go to another place for consultation.(ID7, Doctor with specialty training in Community medicine working at family health centre)* |
|  |  | *Changing their (HCPs) attitude with one class is tough. It will not change because that attitude develops based on this person's experience over the years, his thoughts, and desires. That can never be changed with 1- or 2-day training. We can provoke thought that he can see this topic (mental health issues) like this also, right? Rest, they have to think by themselves. (ID28, Specialist, Psychiatry)* |
|  | Multimorbidity patient characteristics | *Many patients show reluctance to take the medicines we suggest. For example, in diabetes, the patient will consume everything without proper diet control and even do not take medication for it. And when the blood glucose level increases, even if we ask them to take the additional medicines properly for at least two weeks, they complain that they already take many medicines and do not want to add them. (ID4, Doctor working at the family health centre)* |
|  |  | *I still remember a patient, for whom I spend around 10 minutes explaining how important is to adhere to the treatment and medication. In addition, what are the complications that can develop if they did not take the medication? Still, the patient did not take medicines properly and he visited when his condition was worse. This age group (below 40 years) will not accept that they are sick. (ID1, Doctor working at family health centre)* |
|  |  | *Sometimes when the lab report shows a low cholesterol level then the patient automatically stops the medicine without even the consent of the doctor, which becomes a big threat. (ID6, Doctor working at family health centre)* |
|  |  | *I think that the main thing is people will take medicines, but they find it very difficult to make lifestyle changes and modifications. Another thing I have noticed is that people are stressed. So, I would say difficulty in changing lifestyle and stress. (ID2, Doctor with specialty training in Paediatrics working at family health centre)* |

Box S1: Topic guides

| **Topic guide for healthcare providers in the primary care centres**   - Could you please give a brief description of your current role in management of patients in Family Health Centre (FHC)?   *Probes*  *Job title and role*  *Length of time in role, experience, and key responsibilities*   - Could you please describe the organization of care for people who receive treatment from FHC?   *Probes*  *Processes at FHC*   - In your experience, what are the kinds of multiple long-term conditions people present with in your outpatient settings?   *Probes*  *Have you observed any patterns in the types of MM conditions*  *Any difference in patterns of MM among people visiting FHC*   - Could you please describe how you manage people with MM in your centre?   *Probes*  *Availability of protocols/guidelines; what do you do currently do if there are no protocols available?*   - Could you please describe how the health system is currently organized to manage people with multiple long-term conditions?   *Probes*  *Any specific programmes (screening, management, follow-up); If so, how?*  *How does associated programmes such as respiratory conditions help (or not) with the management of MM?*  *How has upgrading to FHC help (or not) with the management of people with MM?*   - Could you please describe some of the challenges you face when you are managing people with MM at your centre?   *Probes*  *Of the several challenges which do you find the most difficult and why*   - Could you describe a situation where you think care at FHC worked well for a patient with MM?   *Probes*  *Why do you think this worked?*   - In your experience with managing people with MM, what have they reported as challenges for them in managing MM? - In your opinion, how should the care for people with multiple conditions organised?   *Probes*  *Role of FHC, health system, family members*  **Topic guide for specialists**   - Could you please give a brief description of your current role?   *Probes*  *Years of experience, any primary care experience, public health system experience*   - Could you please tell me about the type of multi-morbidity conditions you frequently see in your outpatient department?   (If they respond they do not see multimorbidity/ ask for co-morbid conditions)  *Probes*  *Why do they think there is an increase in multimorbidity (if they respond as MM is increasing; reassure there is no right or wrong answer, their opinion)*  *How do they manage patients with MM? (focusing on difference between single condition vs multiple condition)*   - Could you please describe in your experience, what are some of the challenges faced in managing patients with MM? - What do you think would be some of the challenges for HCPs at FHCs in managing patients with MM?   *Probes*  *Lack of guidelines for patients with MM (if they see this as not a problem; ask for reasons)*  *Patient related challenges (difficulties in communicating previous treatment, medication adherence issues, lifestyle management issues etc.)*  *Multiple medicines (lack of combined medicines in public health system-as highlighted by medical officers)*  *Care co-ordination issues (patients visiting multiple providers, mismanaged referrals etc.)*   - Based on your experience what are some of the suggestions for improving care for patients with MM? (specifically in primary care/FHC level)   *Probes*  *Additional screening activities (If so, what conditions and why)*  *Specialist visits at FHC*  *Mental health support by other health workers (supportive psychotherapy)* |
| --- |

Table S2:**Standards for Reporting Qualitative Research (SRQR)***

|  | **Standards for Reporting Qualitative Research (SRQR)*** |  |
| --- | --- | --- |
|  | <http://www.equator-network.org/reporting-guidelines/srqr/> |  |
|  |  | **Page/line no(s).** |
| **Title and abstract** | |  |
|  | **Title** - Concise description of the nature and topic of the study Identifying the study as qualitative or indicating the approach (e.g., ethnography, grounded theory) or data collection methods (e.g., interview, focus group) is recommended | 1 |
|  | **Abstract -** Summary of key elements of the study using the abstract format of the intended publication; typically includes background, purpose, methods, results, and conclusions | 2 |
| **Introduction** | |  |
|  | **Problem formulation** - Description and significance of the problem/phenomenon studied; review of relevant theory and empirical work; problem statement | 3-5 |
|  | **Purpose or research questio**n - Purpose of the study and specific objectives or questions | 5 |
| **Methods** | |  |
|  | **Qualitative approach and research paradigm** - Qualitative approach (e.g., ethnography, grounded theory, case study, phenomenology, narrative research) and guiding theory if appropriate; identifying the research paradigm (e.g., postpositivist, constructivist/ interpretivist) is also recommended; rationale** | 5 |
|  | **Researcher characteristics and reflexivity** - Researchers’ characteristics that may influence the research, including personal attributes, qualifications/experience, relationship with participants, assumptions, and/or presuppositions; potential or actual interaction between researchers’ characteristics and the research questions, approach, methods, results, and/or transferability | 7-8 |
|  | **Context** - Setting/site and salient contextual factors; rationale** | 4-5 |
|  | **Sampling strategy** - How and why research participants, documents, or events were selected; criteria for deciding when no further sampling was necessary (e.g., sampling saturation); rationale** | 6-7 |
|  | **Ethical issues pertaining to human subjects** - Documentation of approval by an appropriate ethics review board and participant consent, or explanation for lack thereof; other confidentiality and data security issues | 8 |
|  | **Data collection methods** - Types of data collected; details of data collection procedures including (as appropriate) start and stop dates of data collection and analysis, iterative process, triangulation of sources/methods, and modification of procedures in response to evolving study findings; rationale** | 7 |
|  | **Data collection instruments and technologies** - Description of instruments (e.g., interview guides, questionnaires) and devices (e.g., audio recorders) used for data collection; if/how the instrument(s) changed over the course of the study | 7, Online supplement –Box S1 topic guides |
|  | **Units of study** - Number and relevant characteristics of participants, documents, or events included in the study; level of participation (could be reported in results) | 7 |
|  | **Data processing** - Methods for processing data prior to and during analysis, including transcription, data entry, data management and security, verification of data integrity, data coding, and anonymization/de-identification of excerpts | 7 |
|  | **Data analysis** - Process by which inferences, themes, etc., were identified and developed, including the researchers involved in data analysis; usually references a specific paradigm or approach; rationale** | 7-8 |
|  | **Techniques to enhance trustworthiness** - Techniques to enhance trustworthiness and credibility of data analysis (e.g., member checking, audit trail, triangulation); rationale** | Online supplement-Box S2 |
| **Results/findings** | |  |
|  | **Synthesis and interpretation** - Main findings (e.g., interpretations, inferences, and themes); might include development of a theory or model, or integration with prior research or theory | 7-10 |
|  | **Links to empirical data** - Evidence (e.g., quotes, field notes, text excerpts, photographs) to substantiate analytic findings | Results section; pages; Online supplement Table S1 |
| **Discussion** | |  |
|  | **Integration with prior work, implications, transferability, and contribution(s) to the field -** Short summary of main findings; explanation of how findings and conclusions connect to, support, elaborate on, or challenge conclusions of earlier scholarship; discussion of scope of application/generalizability; identification of unique contribution(s) to scholarship in a discipline or field | 20-23 |
|  | **Limitations** - Trustworthiness and limitations of findings |  |
| **Other** | |  |
|  | **Conflicts of interest** - Potential sources of influence or perceived influence on study conduct and conclusions; how these were managed | 24 |
|  | **Funding** - Sources of funding and other support; role of funders in data collection, interpretation, and reporting | 24 |
|  | *The authors created the SRQR by searching the literature to identify guidelines, reporting standards, and critical appraisal criteria for qualitative research; reviewing the reference lists of retrieved sources; and contacting experts to gain feedback. The SRQR aims to improve the transparency of all aspects of qualitative research by providing clear standards for reporting qualitative research. |  |
|  | **The rationale should briefly discuss the justification for choosing that theory, approach, method, or technique rather than other options available, the assumptions and limitations implicit in those choices, and how those choices influence study conclusions and transferability. As appropriate, the rationale for several items might be discussed together. |  |
|  | **Reference:** O'Brien BC, Harris IB, Beckman TJ, Reed DA, Cook DA. **Standards for reporting qualitative research: a synthesis of recommendations.** *Academic Medicine*, Vol. 89, No. 9 / Sept 2014  DOI: 10.1097/ACM.0000000000000388 |  |

Box S2: Reflexivity statement

| LTR was a female sociologist, with research expertise and interests in healthcare. LJ was a female, early career researcher with specific interests in intervention development and health systems research. NS and AK were female research assistants with background in nursing and public health. PJ was a researcher with expertise in chronic disease epidemiology and health systems interventions.  Eight out of the twelve authors in this study are Keralites. LJ, LTR, NS, AK and PJ are natives of Kerala. SG is a professor of medical sociology with expertise in cross-cultural research from the UK. The other team members (JVT, MJV, SH, PG, JD and SMH) had expertise in clinical and/ health systems research.  We acknowledge that using Framework Method, the analysis and subsequent themes were influenced by the research team's subjective interpretations of the data. However, throughout the analytical process, researcher reflexivity and audited discussions occurred between authors throughout the data collection, analysis and write-up to ensure rigour in the quality of qualitative analysis conducted. Specifically, charting enabled discussion among the diverse research team and analyst triangulation was ensured, which promoted objectivity between the researcher's position and the analysis. Charting made it easy to identify relevant quotes to illustrate themes from a range of participants and served as an audit trail from raw data to final themes. |
| --- |
